# Chemotherapy Based on Supramolecular Chemistry: A Promising Strategy in Cancer Therapy

**DOI:** 10.3390/pharmaceutics11060292

**Published:** 2019-06-20

**Authors:** Sherif Ashraf Fahmy, Jana Brüßler, Mohamad Alawak, Mayyada M. H. El-Sayed, Udo Bakowsky, Tamer Shoeib

**Affiliations:** 1 Department of Chemistry, American University in Cairo (AUC), AUC Avenue, P.O. Box 74, New Cairo 11835, Egypt; sheriffahmy@aucegypt.edu (S.A.F.); mayyada@aucegypt.edu (M.M.H.E.-S.); 2 Department of Pharmaceutics and Biopharmaceutics, University of Marburg, Robert-Koch-Str. 4, 35037 Marburg, Germany; jana.bruessler@staff.uni-marburg.de (J.B); mohamad.alawak@pharmazie.uni-marburg.de (M.A)

**Keywords:** chemotherapy, supermolecular, anticancer drugs, macrocycles

## Abstract

Chemotherapeutic agents are considered one of the strategies in treating cancer. However, their use is faced by many challenges, such as poor water solubility leading to poor bioavailability and non-selective targeting of cancerous cells leading to diminished therapeutic actions and systemic adverse effects. Many approaches were adopted to overcome these drawbacks and to achieve the targeted delivery of the chemotherapeutic agents to the cancerous cells while minimizing adverse effects. Recently, supramolecular systems such as macrocycles have gained attention in the field of cancer therapy for being able to encapsulate different anticancer drugs via either host-guest complexation or self-assembly leading to a myriad of advantages. This review highlights the most recent studies concerned with the design of such novel systems for cancer therapy.

## 1. Introduction

One of the main challenges in designing drug delivery approaches is selectively delivering drugs to their target cells with minimum adverse effects. In this regard, various drug delivery systems and nano-carriers have been developed, such as liposomes, micelles, polymeric nanoparticles and dendrimers. Despite the effort exerted in developing novel anticancer drugs for effective and safe cancer treatment, targeted drug delivery systems still suffer from serious side effects and limited applicability due to drug hydrophobicity, instability and resistance [1]. However, supramolecular approaches, including cavitands such as calix[n]arenes (CXs), cyclodextrins (CDs), cucurbiturils (CBs) and pillararenes, have gained recent attention as promising alternatives to overcome these drawbacks [2,3]. Macrocycles can act as possible vehicles for different anticancer drugs by either host-guest complexation with anticancer drugs or self-assembly of macrocycles forming drug loaded nano-capsules. This renders their application in clinical studies possible as they typically offer numerous benefits. For instance, the formation of host-guest complexes was found to remarkably improve the water solubility and bioavailability of hydrophobic anticancer drugs in physiological media [1]. The binding affinity of the guest to the host molecules can be changed by adjusting the conditions of the guest’s surrounding environment allowing for better control over the release of the anticancer drug guest inside the cancer cells [1,2,3]. Supramolecular self-assembly could also enhance the targeting of a chemotherapeutic agent to cancer tissue leading to improved anticancer activities with diminished systemic adverse effects [1]. Here, we review the state-of-the-art in this area, with the aim of highlighting the biomedical applications of four major macrocyclic host molecules; calixarenes (CXs), cyclodextrins (CDs), cucurbit[n]urils (CBs) and pillararenes, and their role in cancer therapy. In addition, future research directions for the use of macrocylic host molecules in cancer therapy will be proposed, with the aim of drawing attention for promising potential of utilizing supramolecular chemistry in chemotherapy.

## 2. Macrocycles Host Molecules in Drug Design and Drug Delivery

### 2.1. Overview of Macrocycles

Macrocyclic host molecules are supramolecular systems ranging from 0.3–1.17 nm for internal diameter, 0.496–1.69 nm for external diameter and 0.78–2.24 nm for depth [4,5]. Macrocycles could be spontaneously self-assembled via non-covalent bonding, such as hydrophobic interactions, electrostatic interactions, π–π stacking interactions, hydrogen bonding and van der Waals forces, [6,7,8] which render their reversible dissociation and re-construction feasible at relatively low energy [9]. They have been chosen as promising candidates for drug delivery due to their biocompatible, non-toxic and environmentally-friendly nature [10] in addition to their ability to form inclusion complexes with different guest drug molecules, which, in turn, can enhance the current methods of targeted drug delivery (TDD) in general and targeted cancer therapy, in particular. Over the past few years, some studies reported on the use of macrocycles host molecules, such as cyclodextrins (CDs), pillararenes, cucubiturils (CBs) and calixarenes (CXs), in the efficient delivery of different chemotherapeutic agents. Macrocycles act as host molecules by encapsulating different guest molecules such as small organic molecules, sugars, amino acids within their cavities forming inclusion complexes. This guest-host complexation could involve a combination of hydrophobic, hydrogen bonding, charge transfer, covalent bonding or ion-dipole interactions. [11,12].

**Figure 1:**
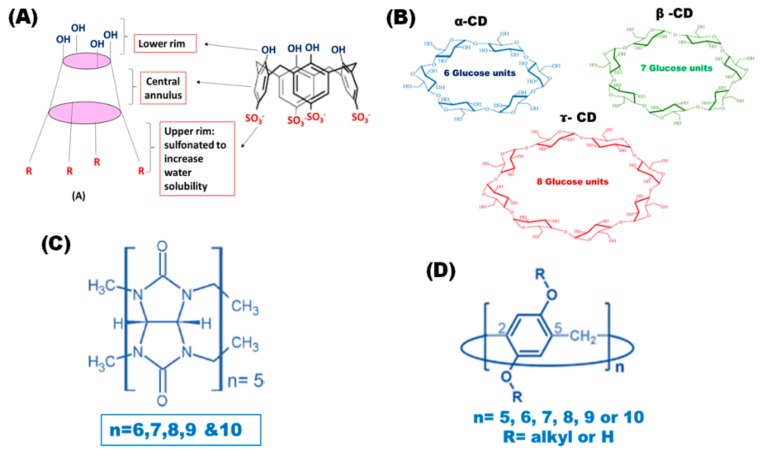
Chemical structure of calixarenes (CXs) and sulfonated CXs (**A**), cyclodextrins (CDs) (**B**) cucurbiturils (CBs) (**C**) and pillar[n]arenes (**D**).

CXs, as macrocyclic host molecules, have attracted much attention during the past few years. They are synthesized via condensation of phenolic units, which are linked to each other at the meta- position through methylene bridges, and formaldehyde in the presence of inorganic bases. Two major synthetic pathways are involved in the synthesis of CXs. The selective synthesis of CXs containing four, six or eight phenolic rings is controlled by optimizing the choice of solvent, base and reaction temperature [13,14,15,16]. CXs have been successfully used in drug delivery because of their unique structure which comprises; an upper rim with para-substituent of phenolic ring, a lower rim with phenolic hydroxyl group and a hydrophobic core (central annulus), as presented in Figure 1A. These structural features enhance their ability to act as host molecules by encapsulating different therapeutic guest molecules within their cavities [11,12]. CXs are easily functionalized through the insertion of different functional groups at the para-position of their phenolic units such as carboxylates, phosphates, ammonium and sulfonates. The chemical functionalization of CXs helps to enhance their water solubility [15]. Recently, sulfonated CXs with even numbers of phenolic units (*n* = 4, 6 and 8) have been widely used because they are easily synthesized via the single stage pathway, using phenolic units with sulfonate groups at the para-position. Sulfonated CXs are water-soluble, biocompatible and safe to human cells [16] and their in vivo dose can reach up to 0.1 g/kg without observable toxic effects [17]. 

In addition to their applications in drug delivery, functionalized water-soluble CXs (such as sulfonatocalix[n]arenes) have been utilized in drug design for increasing the solubility of water-insoluble drugs, biochemical recognition, bio-imaging, gene delivery and enzymatic activities by acting as host molecules capable of accommodating different organic and inorganic molecules. Some CXs and their derivatives also possess pharmacological activities such as being antiviral, antibacterial, antifungal, anti-thrombotic and anticancer agents [18]. For instance, calixplatin is a recent CX derivative that was synthesized using calix[4]arene and four attached cis-diamineplatinum(II) groups forming a potent anticancer functionalized CX with four platinum (II) centers. Calixplatin was reported to have increased anticancer activities (2–4 folds) against three different types of human cancer cell lines and lower adverse effects in comparison to carboplatin. This was attributed to the novel structure of calixplatin, which offers much higher water solubility, higher stability and increased steric hindrance relative to carboplatin [19]. 

Cyclodextrins (CDs) are another class of macrocyclic host molecules for which researchers have found several applications in the pharmaceutical field [20]. They are produced from the enzymatic hydrolysis of starch through a relatively non-expensive method forming α-1,4 glycosidic bond linked oligosaccharides composed of six or more glucopyranose units. CDs have a cone-like structure with a hydrophobic hollow cavity and they possess different sizes depending on the number of linked glucose units; for example, α, β and γ CDs have 6, 7 and 8 glucose units, respectively, as presented in Figure 1 [21]. Their unique structure where the hydroxyl groups of the glucose units are directed toward the outer surface, while the methinic protons are found inside the cavity, which give rise to a hydrophilic outer surface compatible with aqueous media and a hydrophobic inner cavity. This structure allows the complexation of a wide variety of hydrophobic compounds such as proteins, positively and negatively charged molecules, polymers and small molecules [22,23,24]. CDs are generally biocompatible, non-toxic and fairly water-soluble. However, some commercially available CDs derivatives have even more enhanced water solubility such as hydroxypropyl-*β*-cyclodextrin, hydroxypropyl-*γ*-cyclodextrin, sulfobutyl ether-*β*-cyclodextrin and randomly methylated-*β*-cyclodextrin [20]. By virtue of their structure and characterization, CDs showed significant impact on drug design and development by improving the physicochemical and biological properties of different therapeutic agents.

Cucurbit[n]urils (CBs) also have a significant contribution in the drug delivery of chemotherapeutic agents, as they are biocompatible and safe with a maximum adult human tolerated dose of 200 mg/kg [25]. They are synthesized through the condensation of glycouril units and formaldhyde in the presence of acidic media forming barrel-shaped macrocycles with partially enclosed cavities. Thus, they are formed of repeated glycouril units linked by methylene bridges, as presented in Figure 1 [26]. Based on the number of glycourils units, there are different types of CBs, namely CBs 5–10. In particular, CBs 6, CBs 7 and CBs 8 have cavity sizes of 164, 279 and 479 Å, respectively, which are nearly equal to those of α, β and γ CDs [27,28]. The internal hydrophobic cavity of CBs makes them able to encapsulate both neutral and positively charged therapeutic agents through hydrophobic and ion-dipole interactions, respectively. As the number of glycourils units increases, larger molecules or multiple molecules may be encapsulated. For instance, CB 8 can host two molecules by forming a 2:1 guest-host complex [29]. Pillar[n]arenes were first discovered by Ogoshi et al. [30] and due to their symmetric structure, ease of functionalization and their ability to host different molecules, pillararenes have had some recent applications in cancer drug delivery [31,32]. 

### 2.2. Macrocycles-Anticancer Drug(s) Inclusion Complexes

The unique structures of the different macrocyclic molecules enable them to accommodate various guest molecules (including drugs) by non-covalent inclusion complexation through encapsulating them into their cavities. Few studies reported the impact of guest-host complexation on the improvement of anticancer drugs properties, such as increasing the water solubility of hydrophobic drugs, enhancing their stability, prolonging their half-lives and minimizing their side effects [33]. Several recent studies have shown that complexation of drugs with CXs, CDs and CBs could be efficient in enhancing their water solubility. In one study, it has been reported that the solubility of topotecan (TPT) increased by five-folds, from 1.2 to 6.4 mg/mL, through complexation with sulfunatocalix[4][arene (SC4), leading to better anticancer activity [34]. TPT is used in the treatment of ovarian, cervical and small cell lung cancers through the inhibition of topoisomerase I enzyme; however, it suffers from poor water solubility, which limits its use [34]. The complex was studied using UV, 1H NMR and DSC where the findings revealed the formation of SC4-TPT inclusion complex with a molar ratio of 1:1 and a stability constant calculated using the Scatchard method, was found to be 5.6 × 10^−3^ M^−1^ indicating a stable complex [34]. In another study, the water solubility of lonidamine was augmented by more than 380-fold through complexation of the drug with permethyl *β*-cyclodextrin. Bioavailability and anticancer activities were also improved for the drug that exerts its cytotoxic activity through the inhibition of glycolysis within cancerous cells [35]. *β*-CD was also successfully used to prepare a 1:1 molar ratio inclusion complex with methotrexate via the neutralization method, which involves the interaction between the hydroxyl group of *β*-CD and the amino group in methotrexate. The complex also showed enhanced dissolution rate with a solubility constant of 469.5 mol^−1^, and exhibited better bioavailability along with in vitro anticancer activity against BALBIc mice bearing Ehrlich ascites carcinoma, in comparison to methotrexate alone. The findings of this work suggested the possibility of using methotrexate-*β*-CD inclusion complexes as oral dosage form for cancer treatment [36]. Recently, another study on CDs showed the effect of inclusion complexation on enhancing the physicochemical properties of lapatinib (LAP) [37]. This is a strongly cytotoxic drug administered orally as film-coated tablets, and is effective in the treatment of metastatic breast cancer when used in combination with other anticancer drugs such as capecitabine. Lapatinib exerts its antineoplastic activity through the selective inhibition of tyrosine kinase enzyme leading to the inhibition of the epidermal growth factor receptor and human epidermal growth factor receptor 2 [37]. However, LAP suffers from some drawbacks that might affect its efficiency as an anticancer agent such as its very poor water solubility, moderate bioavailability, high dependence of its bioavailability on the intake of food and pH of the stomach (alkaline media decreases its absorption) and its very high therapeutic effective dose (1250–1500 mg) which interferes with patient compliance. In that study, water solubility, bioavailability and anticancer activity of LAP were shown to be improved through its inclusion complexation with CD [37]. Complexes were investigated using different analytical approaches such as UV, ^1^H- NMR, two-dimensional (2D) NMR and molecular modeling, which revealed that β-CD is the most stable host molecule for LAP in comparison to other CD types, and the estimated stability constant (K_stab_) for 1:1 β-CD-LAP complex was 121 ± 12 M^−1^. Furthermore, phase-solubility studies using four CDs (*β*-CD, (2-hydroxypropyl)-*β*-cyclodextrin, randomly methylated-*β*-cyclodextrin and sulfobutylether-*β*-cyclodextrin (SBE-*β*-CD)) showed that SBE-β-CD was able to improve the water solubility of LAP by more than 600-folds [37]. The effect of using γ-cyclodextrin (γ-CD) as a host molecule in improving the water solubility and bioavailability of picoplatin, a newer generation of Pt-II based anticancer drugs was also recently reported [38]. An inclusion complex was successfully formed between picoplatin and γ-CD with a molar ratio of 1:1 and stability constant of 10.3 M^−1^. The anticancer activities of γ-CD-picoplatin complex were investigated using human lung adenocarcinoma and human breast adenocarcinoma cell lines. It was reported that the cytotoxic activities of γ-CD/picoplatin complex was similar to free picoplatin. This indicates that the formation of γ-CD/picoplatin inclusion complex did not decrease the anticancer activities of picoplatin [38]. 

It was shown that encapsulating the albendazole (ABZ) derivative, (2-methoxyethyl) 5-propylthio-1H-benzimidazole-2-yl carbamate (MEABZ) in CB6, CB7 and CB8 led to an increase in its water solubility from 8 µM to 2 mM in case of CB6 and CB7 and from 8 µM to 9.4 mM in case of CB8 as determined using 1H NMR [39]. ABZ is an anti-parasitic benzimidazole drug which was found to possess remarkable cytotoxic effects against liver cancer, colorectal cancer, paclitaxel resistant leukemia and ovarian cancer. The more potent ABZ derivative exhibited anticancer activities ten times more than that of the parent ABZ against human colorectal cancer cell line (HT-29) and human prostate cancer cell line. Despite its higher cytotoxic activities, its weak water solubility still hindered its clinical application in cancer treatment. However, with those recently published findings, new avenues could be available for the potential use of ABZ derivatives in the design of different potent formulations for cancer therapy [39]. 

Another reported advantage for host-guest complexation is minimizing the side effects of some anticancer drugs. For example, doxorubicin (DOX), which is a broad spectrum drug used in treatment of leukemia, lymphomas, breast, ovarian and lung cancers, is accompanied by many severe side effects such as gastrointestinal toxicity, cardiotoxicity and myelosuppression. DOX exerts its cytotoxic effects through strong binding to the nitrogen base pairs of the polynucleotides leading to the inhibition of synthesis and transcription of polynucleotides. The inclusion complexation of DOX with p-sulfonatocalix[6]arene (SC6) was reported to reduce the side effects of DOX, increase its selectivity towards cancer cells and its potency [40]. This complexation was explained through electrostatic interaction between the negatively charged SC6 and the positively charged DOX at physiological pH (pH 7.4). Interestingly, the molar ratio of DOX to SCX6 was found to be 1:1 for DOX concentration less than 10^−4^ M and 2:1 for DOX concentration higher than 10^−4^ M. The latter ratio was attributed to the formation of DOX dimers through π–π interactions between the planar aromatic rings of DOX at higher concentrations. The SC4-DOX selectivity towards the cancerous cells was attributed to the flexible (non-rigid) structure of SC6, which allows for many conformations of the supramolecule, aiding in the approach of SC6 to the di-nucleic acid helix. This results in the interaction of the neutral side of SC6 (containing OH groups) with the polynucleotide of cancer cells enhancing the transport of DOX to the cancerous cells [40]. 

Oxaliplatin, which is a platinum-based drug used as first line therapy in the treatment of colorectal cancers, is known for its adverse side effects including peripheral neuropathy and myelotoxicity. Encapsulating this drug in different novel monofunctionalized cucurbit[7]uril (CB7) derivatives (such as biotin-CB7) resulted in the reduction of these adverse effects as well as the enhancement of the anticancer activity of the drug. The CB7 synthesized derivative-oxaliplatin was found to kill L1210FR cancer cells at significantly lower concentrations (EC50 = 8 μM) in comparison to CB7—oxaliplatin (EC50 = 76 μM) or free oxaliplatin (EC50 = 188 μM) [41]. The encapsulation of oxaliplatin, which is susceptible to photo degradation, using CB7 not only reduced the drug’s adverse effects, but also increased its stability for more than one year. The encapsulated oxaliplatin also showed a reduced reactivity towards guanosine and L-methionine by 2–3 fold and 15 fold, respectively, thus, CB7 protected oxaliplatin from being hydrolyzed by the attack of sulfur containing peptides and proteins [42]. To further enhance the selectivity of CB7, it was covalently bound to biotin in order to target cancer cells that overexpress biotin receptors. The CB7-biotin derivative had biotin ligands on its convex surface while its cavity was available to host oxaliplatin. Compared to free oxaliplatin, the encapsulated drug was reportedly more efficiently delivered to L1210FR cells [41]. In another recent study, a host guest complexation between oxaliplatin and CB7 (oxaliplatin-CB7) was shown to reduce the cytotoxicity of the encapsulated oxaliplatin to healthy colorectal cells while the anticancer activity to cancerous colorectal cells was enhanced, in comparison to free oxaliplatin. These effects were attributed to the replacement of oxaliplatin from the complex by spermine, which is overexpressed in cancerous media [43].

Cisplatin is another platinum-based anticancer drug in use for more than 40 years, in the treatment of different types of cancers. The treatment with cisplatin is usually accompanied with severe dose-limiting adverse effects and with cancer cell resistance. It was also shown that these effects could be reduced by encapsulating the drug in CBs, as was the case with oxaliplatin, which comes from the same family of anticancer agents. Plumb et al. (2012) were the first researchers to report improvements in the anticancer activity of cisplatin along with reductions in its adverse effects and cancer cell resistance through encapsulating the drug in CB7 forming CB7-cisplatin complexes that are stabilized by means of four hydrogen bonds [44]. Surprisingly, this complex was reported to have negligible effects on the in vitro cytotoxicity of cisplatin in the human ovarian carcinoma cell line A2780 and its cisplatin-resistant sub-lines A2780/cp70 and MCP1. However, a pronounced effect was observed on in vivo cytotoxicity using human tumor xenografts, as the complex was able to slow down the tumor growth in the cisplatin-resistant A2780/cp70 lines in comparison to free cisplatin. The effect of the complex on reducing cell resistance was reported to be achieved through modifying the pharmacokinetic effect of cisplatin in systemic circulation. This is because, when administered at the same dose, the total concentration of CB7-cisplatin complex, which is circulating in the blood stream, over a period of 24 h is much higher than that of free cisplatin. These outcomes were, therefore, very promising in overcoming cisplatin cancer resistance through manipulating the pharmacokinetic action of the platinum drug within the blood stream [44]. It is of note to mention, however, that while that study highlights the advantage of using host-guest complexation in overcoming resistance developed by cancer cells to anticancer drugs, no explicit studies reported on the safety of such complexes.

One of the main concerns in drug delivery is the stability and lifetime of the drugs. Encapsulating drugs through complexation has also been shown to enhance drug stability. It was reported that the stabilities of oxaliplatin and tomozolomide (TMZ) were enhanced upon complexation with CB7 [5,45]. Encapsulating TMZ, an effective anticancer drug against glioblastoma multiforme, within the inner cavity of CB7 increased the stability of the drug within the blood stream leading to the prolongation of its half-life time [45]. In addition, the CB7-TMZ complex had a high ability to penetrate the blood brain barrier, which hinders the passage of many drugs into the brain tissue [5]. This finding should be very promising in delivering other anticancer drugs to brain cells. Electrostatic and van der Waals interactions were shown to be responsible for the stability of several CB complexes (such as CB7-cisplatin and CB6-nedaplatin), as reported in a theoretical study using density functional theory calculations at the B3PW91/LANL2DZ level of theory [46]. 

Recently, some of us investigates the host/guest complexation between 4-sulfocalix[4]arene (SC4) and nedaplatin (ND), a second generation anticancer platinum based drug, for potential use in drug delivery. This complexation was studied using a variety of experimental and theoretical methods. The results suggested the formation of a weak 1:1 complex between SC4 and ND. The stability constant of the complex was experimentally estimated to be 3.6 × 10^4^ M^−1^ using a normalized version of Job’s plot which lies within the range of the stability constants (0.01 × 10^3^–1.7 × 10^5^) M^−1^ previously reported for complexes that are designed for drug delivery between different macrocycles and various neutral guest molecules. The stability constant was calculated using a second technique, HPLC, and was found to be 2.1 × 10^4^ M^−1^, which is in line with that calculated using Job’s plot. The stability of SC4-ND complex in the solution was ascribed to the formation of hydrogen bonds between the oxygen atoms of SC4 moiety and the hydrogen atoms of ND ammonia ligands; an interaction that did not involve the penetration of ND inside the cavity of SC4. The results suggested the possible use of SC4-ND as a system for enhancing the bioavailability of ND, and hence, its effective delivery to cancer cells [47]. The above studies on the complexation of different anticancer drugs with different host molecules are summarized in Table 1.

### 2.3. Self-Assembly of Macrocyclic Molecules

**Figure 2:**
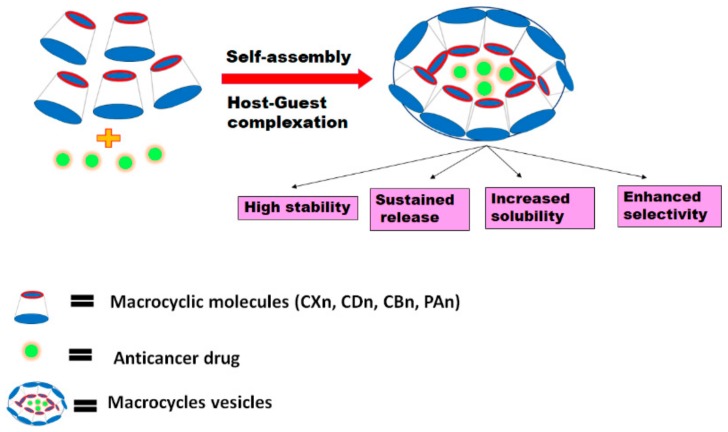
Schematic diagram representing the formation of host-guest vesicles and their impact on anticancer drug delivery.

Some amphiphilic macrocycles can be used to design synthetic vesicles (nanocapsules) and other amphiphilic assemblies based on self-assembly approaches [48]. These assemblies are considered responsive because they are held together by weak and reversible interactions. Thus, their amphiphilic nature enables them to encapsulate both hydrophobic and hydrophilic anticancer drugs within their core shells where inclusion complexation between the host and guest molecules plays a crucial role in linking the hydrophobic and hydrophilic moieties [49]. They have promising applications in the field of cancer therapy because they have a very high water solubility, and can increase the stability of the drugs within the blood stream as well as accommodate different guest molecules through complexation [50]. They were also shown to improve the selective targeting and controlled sustained release actions of the drugs and hence reduce their minimum effective therapeutic doses and adverse effects (Figure 2) [50]. The following is a discussion of the work that has been conducted, to date, on host-guest self -assemblies and their effect on improving the cytotoxic activities and physicochemical properties of different anticancer drugs.

Markowitz et al. (1989) were the first researchers to succeed in designing unilamellar vesicles using CX6 [51]. This was accomplished by injecting tetrahydrofuran solution containing CX6 into water leading to the formation of unilamellar vesicles with size distribution ranging from 0.5–1 µm [51]. Further studies have been conducted on functionalized CX self-assemblies as drug carriers where of the investigated anticancer drugs was paclitaxel (PTX), which is effective against breast, ovarian, lung and colon cancer. Supramolecular nanocapsules of tetrahexyloxy-p-sulfonato calix[4]arene (SC4-C6) were used as novel carriers for PTX. These were prepared by self-assembly of SC4-C6 and encapsulating PTX with a molar ratio of 10 SC4-C6: 1 PTX, respectively; using thin film hydration followed by probe sonication for 30 seconds. The formulated PTX-SC4-C6 nanocapsules had an average particle size of about 206 nm, % encapsulation efficiency (%EE) of about 82.65 % and sustained release rates of loaded PTX. At pH 7.4, about 24.8%, 63.0% and 82.9% of the encapsulated PTX were released at 4, 24 and 72 h, respectively as opposed to rapid release rates of the unloaded PTX of 70% and 85% at 4 and 24 h, respectively. Furthermore, PTX-SC4-C6 showed much stronger cytotoxic activities on human cervical cancer cells in comparison to the free PTX, at concentrations of 1, 10 and 100 µg/mL. It was thus concluded that encapsulating PTX in amphiphilic calixarenes enhanced both release and anticancer properties of the drug [52]. 

Another study reported the encapsulation of PTX into phosphonated, self-assembled calix[4]arene (PCX4) nanovesicles. PCX4 is amphiphilic in nature possessing polar phosphonate head groups and non-polar tail groups resembling the structure of phospholipids which constitute animal and human cells. The PTX-PCX4 nanovesicles were prepared by the thin film hydration method, with a PCX4:PTX molar ratio of 4:1, followed by sonication and were conjugated with long chain polyethyleneglycol (PEG) and folic acid as cancer targeting agents aiming to enhance the selective targeted delivery of PTX. Being pH responsive agents, these nanovesicles had the ability to release the encapsulated PTX in slightly acidic medium; hence, PTX was selectively released in cancerous cells only, since they have a pH of about 5.5 while normal cells have pH of about 7.4. The nanovesicles had an average size of 112 ± 8 nm, zeta potential of −38.76 ± 3.94 mV and %EE of 90.21 ± 4.84%. Release studies revealed that the percent release of PTX in cancerous cells was 75% at 24 h and 85% at 48 h, while the percent release of PTX in normal cells was found to be 20% at 48 h. This indicates that nanovesicles are stable in the systemic circulation (pH 7.4). However, upon reaching cancer cells (pH 5.5), PTX was released, and hence exerted cytotoxic activity. Furthermore, PTX-PCX4 nanovesicles conjugated with folic acid showed larger cytotoxic activity against ovarian cancer cell lines than untargeted PTX-PCX4 vesicles by more than 337% (4 times), demonstrating the positive impact of adding folic acid as a targeting moiety [53]. Table 2 summarizes the major differences between PTX loaded in SC4-C6 and that loaded in PCX4.

A recent study reported the use of amphiphilic sulfonatcalix[4]arene (SC4) as a drug chaperone, for escorting the cationic anticancer drugs mitoxantrone (MTX) and irinotecan (IRC) HCl to the targeted cancer cells based on the co-assembling method [54]. SCX4 is comprised of hydrophilic sulfonate groups at the upper rims and hydrophobic n-hexyl chains at the lower rims, while the critical micelle concentration needed by SCX4 to form a micellar assembly is about 0.5 mM. Co-assemblies of MTX-SCX4 and IRC-SCX4 were prepared by assembling the drugs with SCX4 in molar ratios of 1:4 and 1:1.82 for IRC:SCX4 and MTX:SCX4, respectively; via electrostatic interactions between the tetra-anionic SCX4 and either of the mono-cationic IRC, or the di-cationic MTX. The SCX4-drugs co-assemblies were subsequently surface-functionalized with the targeting ligands of biotin-pyridinium (BtPy) and hyaluronic acid pyridinium (HAPy) for the MTX-SCX4 and IRC-SCX4 co-assemblies, respectively, while bis-MV was used as a cross-linker. Characteristics of these co-assemblies, as well as their anticancer properties, are summarized in Table 3.

Table 3 shows the increase in zeta potential of BtPy-MTX-SCX4 and the decrease in that of HAPy-IRC-SCX4 relative to their non-functionalized counterparts indicating that BtPy and HAPy were successfully introduced on the surface of MTX-SCX4 and IRC-SCX4 co-assemblies through electrostatic compensation between the head groups carrying opposite charges leading to host/guest interaction and non-covalent bonding, respectively. However, the introduction of targeting groups affected neither the average size nor the loading efficiency of the co-assemblies demonstrating the successful additions to the surfaces in a non-destructive and non-covalent manner. These functionalized co-assemblies hold much promise for future cancer therapy, not only for their enhanced anticancer activities, but also for their ability to deliver the drugs selectively to the cancer cells while protecting them from premature degradation [54]. This selective delivery was reportedly achieved by means of passive accumulation due to enhanced permeability and retention (EPR) into the leaky vasculature of cancerous cells [54]. This EPR effect which is important for passive targeting could be attributed to various reasons. First of all, the blood vasculature of normal tissues being organized and closely packed preventing the extravasation of macromolecules is different from that of cancer tissues, which is characterized by being unorganized due to the rapid proliferation of the vascular endothelium, thus producing defective leaky vasculature with high number of open junctions. Secondly, impairment in the lymphatic drainage takes place in tumor tissues, and permeability mediators such as bradykinin, nitric oxide and prostaglandins are overexpressed. This results in an enhanced permeability and retention of the circulating macromolecules. Most clinically used chemotherapeutic agents have low molecular weight and rapidly pass outside the membranes of cancerous cells into the systemic circulation via diffusion, causing poor selectivity and many systemic toxic effects. Hence, drug delivery based on EPR effect is currently found to be the most effective way to selectively deliver anticancer drugs from the macromolecular host molecules into the cancer cells [55,56,57].

The use of self-assemblies of CDs for encapsulating PTX was reported where the drug was encapsulated in mono[6-(2-aminoethyleneamino)-6-deoxy]-β-cyclodextrin (ED-β-CD) forming supramolecular amphiphiles which were subsequently self- assembled into nanovesicles in aqueous medium. These vesicles released their content of PTX upon the external addition of copper ions due to a possible coordination between copper and ED-β-CD which, in turn, reduced the inclusion space and weakened the hydrogen bonding between the host and guest molecules. ED-β-CD-PTX vesicles were prepared by simple addition of saturated ethanolic solution of PTX to aqueous solution of β-CD, with ED-β-CD: PTX molar ratio of 1:1, followed by sonication for 30 min, and finally filtration. The obtained vesicles had an average size of 191 nm and a zeta potential of −22.7 ± 1.3 mV, which indicates an outstanding thermodynamic stability. FT-IR findings revealed that hydrogen bonding was involved in the interaction between the host and guest molecules. Thermal analysis, using thermo-gravimetric analysis and differential scanning calorimetry, confirmed the presence of host/guest interaction as evidenced by the endothermic peak shown for the supramolecular complex as opposed to the exothermic peak displayed for a physical mixture of PTX and (ED-β-CD). This study highlighted the role of introducing copper on releasing PTX from the vesicles that might have a remarkable impact on the development of novel controlled release PTX formulations, since copper exists inside the body in the form of cytochrome C oxidase, superoxide dismutase and tyrosinase [58]. In a similar study, PTX was encapsulated within hyaluronic acid modified β-CD, with molar ratio of 1:1, which was further self-assembled forming biodegradable, biocompatible nanocapsules potent against breast cancer [59]. A particular type of CD-modified self-assemblies, known as polyrotaxanes (PX, cinnamic-acid -modified-α-CD/PEG), was used as vehicles for drug delivery. Two studies were reported in that regard; the first was concerned with delivering methotrexate (MT) drug, which acts as antimetabolite that inhibits the metabolism of folic acid inside the cancerous cells, while the second focused on delivering DOX. In both studies, PX nanoparticles (NPs) were self-assembled, and then the relevant drug was loaded via dialysis followed by freeze-drying. PXs could be promising candidates as drug vehicles, since they are biocompatible and comprise the FDA-approved CD and PEG. They also have remarkable anticancer and release properties. For PX-MT NPs, the average size was about 150 nm, the loading content was 20%, the %EE of the MT was 57% and the formulation demonstrated fast release of MT with % cumulative release of up to 96%. In addition, the PT-MT NPs had a potent cytotoxic activity against HepG2 cells, where the half-maximal inhibitory concentration (IC50) was found to be 5.5 ng/mL [38]. PX-DOX NPs, on the other hand, had an average size of 107 nm, a drug loading content of 18.4% (which is much higher than most polymeric micelles) and %EE of 78.1 %. This formulation showed a burst release of 40% in the first 2 h (in phosphate buffer saline solution, PBS), followed by sustained release of up to 80% at 32 h, as opposed to free DOX which showed a very rapid release of 100% in the first 3 h. PX-DOX also demonstrated a potent anticancer activity towards breast cancer cells in a mouse model and the tumor inhibition rate was found to be 53% which exceeded that of free DOX [60]. This is because the internalization of PX-DOX into cancer cells did not occur due to diffusion, on the contrary to free DOX; hence, the endocytosis of PX-DOX was slower than that of the free drug. Furthermore, the cardiotoxicity of DOX was dramatically reduced after it has been encapsulated into the PX NPs [38,60].

Recent studies showed the potential effect of pillar[n]arenes-based supramolecular vesicles on the efficient targeted delivery of different anticancer drugs. The first attempt to design acid responsive self-assembled micelles based on the host/guest interaction between a novel DOX based prodrug (DBP) and water soluble pillar[6]arene (WP6) was reported by [61]. DOX based prodrug was synthesized by the conjugation of isoniazide (pyridine derivative) to DOX through an acid cleavable hydrazine bond. Afterwards, the WP6-DBP micelles were prepared by the injection method. The prepared micelles had an average size of about 74.6 nm and a spherical micellar structure, which allowed them to passively target cancerous cells. The release study revealed that WP6-DBP supramolecular micelles were stable under normal physiological conditions (pH 7.4), where the percent cumulative release of DOX was found to be less than 10% in the first four hours. However, the prepared micelles showed very rapid release of DOX under acidic conditions (pH 5.5 of cancerous cells) with a percent cumulative release of 100% in the first 30 min. This is because the hydrazine bond in the DOX prodrug was cleaved in the acidic medium resulting in the quick and selective release of DOX inside the acidic cancerous cells. It was also reported that the WP6-DBP micelles were able to enter SKOV3 cancer cells through endocytosis, a finding that may pave new avenues in cancer treatment [61].

The design of multi-responsive supramolecular binary vesicles based on the host/guest interaction between WP6 and DOX was recently reported [62]. The release of DOX from the vesicles was controlled by either adjusting the pH or the introduction of calcium. DOX loaded vesicles had an average size of 190 nm which increased by more than 10-fold upon addition of calcium chloride due to fusion of the vesicles and their subsequent disruption leading to drug release in the desired cell. Release of DOX from this smart controlled release system increased from 3% to 43% in the first 20 min, upon addition of 0.1–5 mM calcium chloride, while cumulative release in the first 5 h was 37% at pH 5, 50 % at pH 3.5 and 5% at pH 7.4. Consequently, DOX was selectively released in cancerous cells possessing acidic pH, while its release was hindered in normal tissues. Cytotoxicity investigations for DOX loaded vesicles revealed that their anticancer activity was the same as that of the free drug. However, it was observed that cytotoxic effects for normal cells are lower than those for free DOX [62]. The advantage of using this multi-responsive system over the PX-DOX system mentioned earlier is the ability of controlling the release by means of pH and CaCl_2_; however, the latter system is more efficient in tumor inhibition relative to free DOX. Table 4 summarizes the major differences between PX-DOX and WP6-DOX.

**Figure 3:**
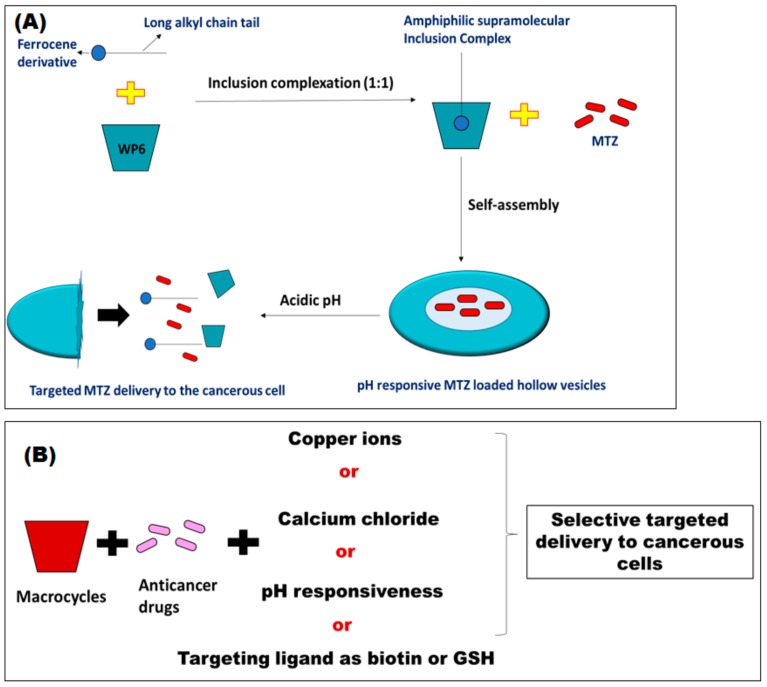
(**A**) Schematic diagram representing the design of pH responsive mitoxantrone (MTZ) loaded supramolecular vesicles, (**B**) Host-Guest responsive complexes. GSH: glutathione.

The pillararene WP6 was also utilized in conjunction with a ferrocene derivative that has a long alkyl chain, in order to form a water-soluble inclusion complex with WP6 through a molar ratio of 1:1. This complex further self-assembles into pH responsive, hollow supramolecular vesicles in aqueous medium. These vesicles were used to encapsulate the hydrophilic anticancer drug mitoxantrone (MTZ) (Figure 3A). Despite its low % EE of MTZ (11.2%), MTZ was efficiently released in acidic medium exerting the same cytotoxic activity as free MTZ while its toxic effects on healthy cells were lower than those of free MTZ [63]. A very recent study reported on the design of dual acid responsive supramolecular system for efficient delivery of DOX [64]. This system was constructed by the host-guest interaction between pillar[5]arene (WP5) and 2,4,8,10-tetraoxaspiro[5.5]endecane moiety (acid-sensitive guest molecule) forming hollow macrocyclic vesicles. The dual acid responsiveness of this system was attributed to the pH response of WP5 in addition to that of the guest molecule. DOX has, in turn been loaded in the stern region of the hollow vesicles. When the DOX loaded vesicles reached the acidic environment of cancer cells, they were cleaved with the help of both WP5 and the acid-sensitive guest molecule resulting in much faster and efficient release of DOX (which reached 91 %) selectively inside the cancer cells. Furthermore, this system showed a pronounced cytotoxic activity against MCF-7 (human breast adenocarcinoma), U87MG (human primary glioblastoma), and HepG2 (human liver carcinoma) cancer cells compared to free DOX. This improvement in the anticancer activity is because the DOX loaded supramolecular vesicles entered cancer cells by endocytosis, resulting in a pronounced DOX accumulation within cancer cells [64]. 

In addition to pH responsive supramolecular vesicles, glutathione (GSH) responsive ones were designed [65]. These can respond to the high concentration of GSH within the cancerous cells, which is much higher than that in normal cells. In that regard, MTZ loaded vesicles that are both pH and GSH responsive were synthesized via host/guest inclusion complexation between WP5 and a lysine derivative containing a disulfide bond. MTZ was then encapsulated in the self-assembled vesicles, which were further disassembled releasing their content of MTZ in the cancerous cells due to cleavage of the disulfide bond in presence of high GSH concentration. These vesicles showed potent anticancer activities particularly in inhibiting the proliferation of HepG2 cancer cells [65]. The different methods proposed for the design of responsive host/guest molecules that deliver cytotoxic agents selectively to the cancerous cells are summarized in Figure 3B.

### 2.4. Host-Guest Systems under Clinical Trials

Since the use of macrocylic host molecules in cancer therapy is relatively recent, only one host-guest chemotherapeutic formulation has been clinically investigated. CRLX101 is a host-guest formulation, which is currently under phase 1/2a clinical trial [66]. CRLX101 is composed of camptothecin (CPT) anticancer drug covalently bonded to β-CD/polyethylene glycol co-polymer and self-assembled. CRLX101 was found to enhance the water solubility of CPT, thus improving its pharmacokinetics, bioavailability and increasing its in vivo systemic circulation enabling the drug to invade solid cancers via leaky blood vessels [66].

## 3. Future Prospects and Challenges

Despite the endeavors in developing novel host-guest systems for the aim of cancer treatment, many challenges do exist, which need further research. Among these challenges is the lack of sufficient knowledge on the biodegradation of supramolecular systems, their immunological reactions, metabolism, excretion and long-term effects. Future studies should address the safety issues related to the use supramolecular systems in chemotherapy, rather than focusing only on their fabrication process. In-depth studies on in vivo models would add a wealth of information regarding the clinical applications and trials of these systems in the hope of finding new effective cancer therapies. Future work should also address supramolecular systems that aim at the diagnosis and imaging of tumors. These systems, when successful, will help in the accurate determination of tumor location and progress rate, and hence, aid in specifying the treatment of choice. 

## 4. Conclusions

In this review, we summarized the most recent state-of-the-art studies regarding the use of supramolecular systems as possible vehicles for many chemotherapeutic agents. The ability of macrocycles to act as host molecules by encapsulating different guest molecules within their cavities, forming inclusion complexes, was discussed. Amphiphilic macrocycles were shown to be successfully used to encapsulate different agents based on self-assembly approaches whereby the produced responsive assemblies are held together by weak and reversible interactions. Reported studies have shown that macrocycles have a very high water solubility, and can increase the stability of the drugs within the blood stream as well as accommodate different guest molecules through complexation. Macrocycles were also found to improve the selective targeting and controlled sustained release actions of a variety of drugs, and hence, reduce their minimum effective therapeutic doses and adverse effects. This is due to macrocycles enhancing the water solubility of anticancer drugs, which thus improve their pharmacokinetic profiles and bioavailability. Anticancer drugs based on supramolecular systems were shown in many cases to have more pronounced anticancer activity compared to their free drugs analogues most likely due to enhanced permeation and retention effects. These systems are therefore likely promising candidates for cancer therapy.

## Figures and Tables

**Table 1:** Examples for host-guest complexes with enhanced anticancer activities and physicochemical properties.

Host	Guest	Targeted Cancer Type	Advantage	Ref.
**SC4**	Topotecan (TPT)	Ovarian, cervical and small cell lung cancers	Increasing water solubility and anticancer activities	[34]
**SC4**	Nedaplatin	Head, neck, lung, testicular and cervical cancers	Enhancing bioavailability and cancer cell delivery	[47]
**SC6**	Doxorubicin (DOX)	Leukemia, lymphomas, breast, ovarian and lung cancers	Enhancing selectivity and reducing adverse effects	[40]
**β-CD**	Lapatinib (LAP)	Metastatic breast cancer	Improving the water solubility, bioavailability and anticancer activity	[37]
**PM-β-CD**	Lonidamine	Prostatic cancer	Increasing water solubility	[35]
**β-CD**	Methotrexate	Melanoma	Increasing solubility and bioavailability and enhancing antitumor activity.	[36]
**γ-CD**	Picoplatin	Lung and breast cancer	Improving water solubility and bioavailability	[38]
**CB6**	Nedaplatin	Head, neck, lung and cervical cancers	Increasing complex stability	[46]
**CB6** **CB7** **CB8**	MEABZ (Albendazole derivative)	Colorectal and prostatic cancer	Dramatically increasing water solubility	[39]
**Biotin CB7**	Oxaliplatin	Colorectal cancer	Decreasing adverse effects and minimizing the therapeutic dose	[41]
**CB7**	Oxaliplatin	Colorectal cancer	Enhancing the stability and reducing adverse effects	[42]
**CB7**	Oxaliplatin	Colorectal cancer	Increasing selectivity	[45]
**CB7**	Oxaliplatin	Colorectal cancer	Reducing toxicity to normal cells and enhancing the anticancer effects to cancerous cells	[43]
**CB7**	Cisplatin	Broad spectrum anticancer activity	Overcoming cancer cell resistance and reducing adverse effects	[44]
**CB7**	Tomozolomide	Glioblastoma multiforme	Prolonging life time and enhancing the blood brain barrier permeability	[5]

**Table 2:** The differences between paclitaxel- tetrahexyloxy-p-sulfonato calix[4]arene (PTX-SC4-C6) and paclitaxel- phosphonated calix[4]arene (PTX-PCX4) in terms of pH responsiveness, average particle size, %EE and % release rates at 24 h.

	PTX-SC4-C6 [52]	PTX-PCX4 [53]
**pH Responsiveness**	None	pH-responsive
**Average Particle Size (nm)**	206	112
**%EE**	82.65	90.21
**% Release Rate at 24 h**	63.0 (pH 7.4)	75% (pH 5.5) not comparable

**Table 3:** Average sizes, zeta potentials and loading efficiencies of the prepared SCX4-drugs co-assemblies [54].

	IRC-SCX4	MTX-SCX4	HAPy-IRC-SCX4	BtPy-MTX-SCX4
**Average size (nm)**	173	234	173	234
**Zeta potential (mV)**	−20	−35	−30	−21
**% loading efficiency**	65.2	43.0	65.2	43.0
**% cell viability (MCF-7 cell line)**	30	18	>10	< 10

**Table 4:** Differences between polyrotaxanes (PX)-DOX and water soluble pillar[6]arene (WP6)-DOX in terms of controlled release, size, %EE, % release and % tumor inhibition. PBS: phosphate buffer saline solution.

	PX-DOX [60]	WP6-DOX [62]
**Controlled release**	None	Multi-responsive (controlled by pH and CaCl_2_)
**Average particle** **size (nm)**	107	190 (increased by adding CaCl_2_ which led to particle disruption in cancerous cells)
**% Release**	Burst release: 40% in the first 2 h. Sustained release: up to 80% at 32 h, neutral pH (PBS)	3% to 43% in the first 20 min, upon addition of 0.1–5 mM CaCl_2_. Cumulative release in the first 5 h: 37% at pH 5, 50 % at pH 3.5 and 5% at pH 7.4.
**%Tumor inhibition**	53% more than free DOX	Same as free DOX
